# Perception of addictions and religiosity in medical students

**DOI:** 10.1192/j.eurpsy.2022.2130

**Published:** 2022-09-01

**Authors:** A.S. Ellouze, M. Maalej, R. Feki, I. Gassara, N. Smaoui, S. Omri, L. Zouari, M. Maalej, N. Charfi, J. Ben Thabet

**Affiliations:** Hedi Chaker University Hospital, Psychiatry C, Sfax, Tunisia

**Keywords:** Perception, religiosity, medical students, addictions

## Abstract

**Introduction:**

Religiosity is among the factors that determine the doctor’s relationship with his addict patient and the empathy he should have.

**Objectives:**

To verify whether future doctors are aware of the addictogenic power of certain substances and certain behaviors and to study their perception of different addictions according to religiosity.

**Methods:**

This was a cross-sectional study with interns and externs of the Sfax medical faculty, in November 2016, via an anonymous questionnaire.

**Results:**

141 students were included and 98,6% declared to be believers. The average age was 23 years. The sex ratio was 0.38. Alcoholism was the addiction most considered as a sin (87.9%), smoking 51.8%, hookah 45.4%, cannabis 78%, gambling 77.3%, internet addiction 16.3%, video game addiction 15.6%, work addiction 8.5%, and exercise addiction 5.7%. Female gender was more often correlated with perceived alcoholism, cannabis addiction, and gambling as sins (p = 0.002; p <0.001 and p = 0.043, respectively). Gambling was significantly more condemned by the participants who fasted (p <0.001). Prayer was significantly correlated with religious disapproval of addictions to tobacco, hookah, alcohol, cannabis and gambling (respectively p <0.001, p = 0.001, p <0.001 , p <0.001, p <0.001). Smoking, hookah and alcohol were significantly more perceived as sins by veiled women (respectively p = 0.011, p = 0.002, p = 0.040).

**Conclusions:**

According to our study, most medical students have a religiously hostile attitude to many addictions. Improving medical training in addictology would allow them to adopt the necessary empathic attitude, without being judgmental.

**Disclosure:**

No significant relationships.

